# Epidemiology of Viral Hepatitis and Liver Diseases in Cambodia

**DOI:** 10.5005/jp-journals-10018-1125

**Published:** 2015-01-06

**Authors:** Bun Sreng, HOK Kimcheng, LY Sovann, ENG Huot

**Affiliations:** 1 Department of Communicable Disease Control, Ministry of Health, Phnom Penh, Cambodia; 2 National Blood Transfusion Centre, Phnom Penh, Cambodia; 3 Department of Communicable Disease Control, Ministry of Health, Phnom Penh, Cambodia

**Keywords:** Viral hepatitis, Liver cirrhosis, Hepatocellular carcinoma, Vaccination.

## Abstract

In Cambodia, the true burden of viral hepatitis has not been revealed, but many surveys were carried out focusing on specific population or on small scales. Different markers of viral hepatitis were found between 27 and 97% in children and almost 100% in adults. Viral hepatitis B in children was 3.5% in 2006 and dropped in 2011; and in adults, it ranged from 4.5 to 10.8%. Viral hepatitis C was between 0.87 and 14.7%. No data are available for hepatitis D in the country. Viral hepatitis E (anti-HEV IgG) went from 7.2 to 12.7%. The complications due to viral hepatitis including chronic liver diseases and hepatocellular carcinoma were reported in the health information system. Around 79% of the patients with high transaminase had at least one viral marker and about 45% of the adults with chronic hepatitis and liver cirrhosis were positive for Hepatitis B surface antigen (HBsAg). Hepatocellular carcinoma accounted for 19.1% of all reported cancer cases. Hepatitis B surface antigen was found in between 55 and 90% in adults with hepatocellular carcinoma and anti-HCV in one-fourth. The only intervention implemented in Cambodia is vaccination against viral hepatitis B (HepB vaccine).

**How to cite this article:**

Sreng B, Kimcheng HOK, Sovann LY, Huot ENG. Epidemiology of Viral Hepatitis and Liver Diseases in Cambodia. Euroasian J Hepato-Gastroenterol 2015;5(1):30-33.

## INTRODUCTION

Cambodia is situated in the South-East Asia with a population of 13,395,682 and population growth rate is of 1.54% (2008). There are more females than males (51.4% *vs* 48.6%). The majority of Cambodian people depend on the agriculture. The main industry in the country is garment factories that employs hundreds of thousands workers. In Cambodia, communicable diseases remain a major challenge and common causes of morbidity and mortality in the country.^1^ World Health Organization (WHO) estimated that, in global perspective, hundreds of millions people are infected with the viral hepatitis in which 240 million became chronic with hepatitis B and 184 million have antibodies to hepatitis C. Globally, hepatitis B and C account for 57% of cases of liver cirrhosis and 78% of cases of primary liver cancer.

Viral hepatitis is prevalent and poses a major public health concern according to WHO. But, the epidemiology of viral hepatitis is not well understood due to many factors: (1) Lack of the information in the national reporting system, (2) limited capacity of the laboratories, (3) limited access to prevention and control of viral hepatitis B and C, (4) no policy to screen viral hepatitis B and C, except the blood donors and (5) few researches.

The objective of this review is mainly to understand the epidemiology related to viral hepatitis in Cambodia using the available information in the country and on the internet.

## VIRAL HEPATITIS

The epidemiology of the viral hepatitis is not well understood in Cambodia.

## VIRAL HEPATITIS A

The prevalence of anti-HAV IgG was very high in both children (27-97%) and adults (almost 100%) in Takeo province and among Cambodian migrant workers in Thailand respectively.^2,3^ More than half of sick children (55%) presenting clinical hepatitis at the National Pediatric Hospital in Phnom Penh from July 1996 to September 1998 were positive for hepatitis A virus, and hepatitis B virus, but not for hepatitis C or hepatitis E virus.^4^ There have been no documented outbreaks of viral hepatitis A in Cambodia.

## VIRAL HEPATITIS B

The WHO ranked Cambodia in the list of the countries with the prevalence of the hepatitis B is moderate (5 to 7% of the total population).

### Viral Hepatitis B in Children

The seroprevalence of the Hepatitis B surface antigen (HBsAg) among 5-year-old children was 3.5% (95% CI 2.4, 4.8%) in 2006 and has dropped in 2011.^5^

### Viral Hepatitis B in Adults

The prevalence of viral hepatitis ranged from 4.5 up to 10.8% in the different studies in Cambodia and among Cambodian migrant workers in Thailand.^2,6,7^ Similarly, 8.6% of Cambodian women born in Cambodia and live in Australia were positive for HBsAg. Hepatitis B surface antigen was prevalent among the adults with acute hepatitis (46%).^6^ The prevalence of anti-HBc ranged from 38.5% among adults in Siem Reap province to 58.6% among blood donors.^7^ The prevalence of both positive for HBsAg and anti-HBc was 7.3%.^7^ Genotype C represented 86% (99% was C1) followed by genotype B (11.6%).

### Prevalence of Viral Hepatitis B among Patients with HIV/AIDS

Less than 5% of the children infected with human immunodeficiency virus (HIV) attending HIV/AIDS clinic at the National Pediatric Hospital was positive for hepatitis B virus and 11.0 to 12% of adults on antiretroviral (ART) was positive for hepatitis B virus.

## VIRAL HEPATITIS C

World Health Organization estimated that Cambodia has the prevalence of hepatitis C in between 2.0 and 2.9%.^3^ The prevalence of the hepatitis C (anti-HCV positive) ranged from 0.87 to 14.7%.^2,6-8^ The HCV-RNA accounted for 2.3% in Siem Reap province.^6^ Its genotypes were six (54.5%) and 1 (27.3%). In the patients with HIV/AIDS, the prevalence of the anti-HCV and HCV-RNA were 0.9% in children and 62% (31/50) in adults respectively.^8^ The HCV genotype 1 is prevalent (68%) followed by genotype 6 (25%).

## RISK FACTORS FOR VIRAL HEPATITIS B AND C

There were very few studies about the risk factors for getting infected with viral hepatitis B and C in Cambodia. The common risk factors for HIV/AIDS included heterosexual, noncommercial sexual relationships, mother-to-child transmission, surgery, blood transfusion, fibroscopy, injection drug use, and male-to-male sex. The needle injury was also common.^6,9,10^

## VIRAL HEPATITIS D

The prevalence of the hepatitis D has not been known in Cambodia.

## VIRAL HEPATITIS E

The seroprevalence of hepatitis E (anti-HEV IgG) in Cambodia was between 7.2 and 12.7%. Hepatitis E was also found in pigs. Genotypes 1 and 3 were identified.

## LIVER DISEASES

At least 79% patients with increasing transaminase were found to have at least one viral marker, 41% positive for HBsAg and 39% for anti-HCV.^11^ A study by Thuring EG et al revealed that 45% of the adults with chronic hepatitis/ liver cirrhosis was positive for the hepatitis B (HBsAg).^2^ Another study found prevalence of hepatitis B and C was 47.8% (65/136) and 40.8% (42/103) respectively. Almost 40% of the coinfected HIV/HCV patients met clinical, biological, and/or ultrasonographic criteria for cirrhosis.^9^ Other infectious agents that cause liver diseases include amebiasis, schistosomiasis and opisthorchiasis. Other microorganisms can cause liver abscess.

### Hepatocellular Carcinoma

The liver cancer accounted for 19.1°% of all reported cancer cases according to the health information system (HIS) of the Ministry of health (413/2179) in 2013. No specific causes of the cancer were elaborated.^1^ The seroprevalence of HBsAg was between 55.35 and 90% in adults with hepatocellular carcinoma (HCC).^2^ Around one-fourth was positive for anti-HCV (10/40). The co-infected accounted for 7.5% (3/40).

## CONCLUSION

Viral hepatitis B and C in Cambodia is very prevalent (~8%) according to the WHO’s estimation. Cambodia government set up the goal to eliminate the hepatitis B by 2017, through National Immunization Program (NIP). There is no comprehensive policy, strategy and guidelines available for the viral hepatitis in the country.

### Reporting and Surveillance

Currently, the viral hepatitis and liver diseases cannot be made available by HIS of the Ministry of Health. They were reported in the other diseases.^2^ Syndromic surveillance ‘Jaundice’ in the Cambodia Early Warning System (CamEWARN) of the department of communicable disease control (CDC) has been incorporated in which few cases were reported.

### Prevention

In 2013, the coverage of the hepatitis B birth dose (<24 hours) was 60% and the DPT-HepB-Hib3 was 95%. The health education by the National Center for HIV/ AIDS, Dermatology and STD (NCHADS) contributed to the decline of the prevalence and the incidence of the viral hepatitis B and C as well. Anyway, the transmission by blood transfusion still occurs as the quality of the current tests at the blood banks and the practice of the infection prevention and control is still compromised. There is a possibility of the outbreak of the hepatitis A and E in children as it was hygiene/sanitation and evidence found in the river water in Siem Reap Province, Cambodia.^12^

### Treatment

There is no policy to screen people for viral hepatitis. Management of viral hepatitis was included in the National Therapeutical Guideline for Referral Hospital, but only symptomatic care. The antiviral drugs for viral hepatitis are not listed in the national essential drugs of the Ministry of Health.

## WAYS FORWARD

To improve the equity access to the prevention and control of the viral hepatitis in Cambodia, the following should be considered:

- Create a national coordination body to overall coordinate the prevention and control of the viral hepatitis in addition to the NIP and develop the national policies, strategies and guidelines to prevent and control the viral hepatitis in the country.
  –   Enhance surveillance and reporting of viral hepatitis at all the health facilities.
  –   To strengthen the quality of the testing at the national/provincial blood banks (22) by the implementation of the quality control system (QCS) and the external quality assurances (EQA).
  –   Establish satellite care centers for the patients infected with viral hepatitis B and C.
  –   Make the rapid tests available at all referral hospitals and if possible at selected health centers.
  –   Raise awareness of viral hepatitis among the public, especially pregnant women and those at high risk (HIV/AIDS, tuberculosis).
- Increase deliveries at the health facilities and ensure all the newborns receive the hepB birthdose and all received DPT-HepB-Hib3, and consider the immunoglobulin if affordable.
- Mobilize resources to support the prevention and treatment for the viral hepatitis.
- Conduct more researches are needed to better understand the epidemiology of the viral hepatitis, especially the hepatitis A, D, E and its comprehensive prevention and control.
